# Space technologies for health

**DOI:** 10.2471/BLT.15.030815

**Published:** 2015-08-01

## Abstract

Space science and satellite technologies hold untapped potential for public health, according to a new expert group that will deliver its proposals to the United Nations General Assembly in New York next month. Pascal Michel talks to Fiona Fleck.

Q: How are Canada and other countries collaborating with the World Health Organization (WHO) on space technologies and health?

A: Space agencies around the world have a long history of collaboration, including with public health agencies. A key element of our collaboration is the United Nations (UN) Committee on the Peaceful Uses of Outer Space, which was set up by the UN General Assembly in 1959 to govern the exploration and use of space for the general benefit of humanity. This year the UN set up an expert group on space and global health, under the leadership of the Canadian government. WHO is collaborating with Canada on this, through our Canadian Space Agency, as well as other space agencies and international space organizations, including the European Space Agency, the Japan Aerospace Exploration Agency, the National Aeronautics and Space Administration, the Russian Federal Space Agency and the UN Office for Outer Space Affairs.

“Space agencies around the world have a long history of collaboration, including with public health agencies.”

Q: What does the UN expert group on space and global health do?

A: The expert group review and analyse current uses of space – with regard to technology, applications, practices and initiatives – and how these can better serve global public health needs. Their observations are articulated in the context of efforts to use space technology for socioeconomic development and captured in the United Nations Committee on the Peaceful Uses of Outer Space 2015 (UN-COPUOS) report, which will be submitted to the UN General Assembly next month. The expert group will explore potential applications of all the space sciences in global health by seeking collaborative ways of addressing public health problems that are oriented towards the users’ public health needs. The expert group will continue efforts to sustain the active engagement of international partners in this domain. These issues will be discussed at the United Nations Conference on Sustainable Development, where governments are set to adopt a new set of development goals.

Q: How long have we been using space science and satellite technology for public health purposes?

A: Little was known about the effects of space flight on human physiology in the early days of space exploration and nations soon became interested in space medicine. As for satellites, the first accounts of the potential benefits of remote-sensing satellites were documented in the late 1980s, with a notable increase in this focus in the late 1990s, particularly for health issues related to mosquito-borne disease, such as malaria, and to issues related to water and agriculture.

Q: How can the science of space medicine be applied to public health on the Earth?

A: We can benefit from all sorts of medical advances achieved in the preparation of space missions. For example, devices developed for space missions, such as the robotic arm that was used to assemble the international space station, have been adapted into a smaller robotic device for use in surgery or adapted to be used inside a magnetic resonance imaging scanner to assist with cancer diagnostics.

Q: What are some examples of technologies that are applied for health purposes?

A: Different types of satellites are useful for public health. Communications’ satellites enable long-distance telephone or digital communications on Earth in places where there is no telephone infrastructure. One public health application of this is telemedicine or tele-health, which can be used in remote locations and can link health experts with other health professionals or patients anywhere in the world via satellite communications. The other major area is the use of Earth observation satellites, i.e. those that look down at Earth. These can do two things. We may see them as fancy cameras taking photographs, but they are more sophisticated than that as we can use them to analyse the light that bounces off the Earth and thus analyse what we call the “fingerprint” of the land, air or sea. For example, this technology can help measure the wind, clouds or rain or other environmental parameters that are associated with the presence of insects that can carry diseases. This technology can also help us measure the extent a region is affected by drought or flooding and be used for the assessment of food supply or potential for water-borne diseases. In addition, some of these satellites – those that make up the global positioning system (GPS) that is used in mobile phone technology – can also be used to locate precisely certain objects on Earth and, for example, when we are investigating outbreaks we can use GPS technology to pinpoint the location of a suspected source of contamination or the location of infected individuals.

Q: What is remote sensing and how can it be useful for public health?

A: Remote sensing means gathering data on an object from a remote location, such as taking temperature readings of the land surface from a satellite without touching the land. One major benefit of satellite remote sensing is that it generates a similar type of data over a large territory, and over a long period of time, which public health officials can use to monitor and assess large areas of land for their programmes. Satellite remote sensing usually works by measuring the reflection of light bouncing off objects on the Earth, thus we can see, for example, changes in vegetation, humidity in the soil or the type of crop that is growing. Scientists can use these data to locate mosquitoes and predict outbreaks of mosquito-borne disease. Remote sensing can also be used to look at environmental factors for disease, such as water temperature, the air, the type of soil and whether there is dense agriculture, etc. Public health scientists and investigators can use these data to better understand and possibly control factors for contamination or disease transmission. We can also use remote sensing technology to obtain important information we need to respond in an emergency as it can help us to identify, for example, whether there is still a road, a water source or infrastructure.

Q: You have talked about the uses of satellite technology in global public health. How do you use satellite data at home in Canada?

A: At home, our public health agency has used space technology to better understand potential risks arising from water contaminated with infectious disease agents, with a focus on recreational water from medium-sized lakes. Canada has thousands of lakes used for swimming and other recreational activities and it would be very tedious and labour intensive to physically visit and assess each one. We were able to show that satellite data, in combination with other types of information collected, can provide an efficient way to identify potential risk areas that require further investigation. Our public health agency is also using these data to assess the risk of vector-borne diseases, such as Lyme disease and West Nile virus. Given that the ecology of these vectors is linked to climatic and other environmental factors that can be gauged by using satellite data, we are developing new knowledge about ticks and mosquitoes, and new methods to monitor and investigate their habitats.

Q: What is satellite mapping and how can it be used in public health?

A: Broadly speaking, this means using satellite Earth observations and satellite global positioning locations to compile maps. It usually involves integrating these satellite data with other land features (such as roads, buildings and landmarks) and with population characteristics (such as how many people live in a given area) into a geographical information system so that we can visualize the data or perform more advanced spatial analyses. Satellite mapping can be useful to locate hospitals and other health facilities or to assess the best spot to locate a camp for relief workers.

“We expect to see more user-friendly tools to select, analyse and integrate satellite data into epidemiological studies.”

Q: Are data generated by space agencies free of charge?

A: Many satellite data can be acquired free of charge, it depends on the type of data and the intended usage. Some are available publically, some are provided under specific agreement with a space agency or organization in charge of distribution, and other types of data can be obtained for a fee to cover their acquisition and distribution. Under the International Charter on Space and Major Disasters, countries and humanitarian organizations can request and receive satellite data for emergency purposes. The charter was activated during the 2014 Ebola outbreak, to help plan the response effort by locating existing and planned Ebola health-care facilities in West Africa.

Q: What do you foresee in the future of space technology and public health?

A: There are a few exciting things. Ten or 20 years ago, the sensors on board satellites were designed for general use in measuring a few environmental variables, but now space agencies around the world are engaging specific stakeholders and partners, such as public health agencies, to design instruments that are more specifically suited to their needs, so we have more finely-tuned instruments for use in the future. First, the resolution of images generated by observation satellites is improving dramatically in terms of how finely you can see things and how often a set of similar satellites passes over the same location for taking repeated measurements, so going forward we have better and more data. In this way we are, for example, better able to predict disease threats using near real-time plotting of data on maps. Second, we can see some level of democratization of the field itself: 10 or 20 years ago, access to the technology and products was difficult and only highly specialized experts were able to manipulate these products. Now we have come a long way to make the access to and manipulation of these data less technically challenging, opening the opportunity for analysts to apply the technology in public health. This trend may continue in the future and we expect to see more user-friendly tools to select, analyse and integrate satellite data into epidemiological studies.

